# Silver Nanoparticle-Based Sensor for the Selective Detection of Nickel Ions

**DOI:** 10.3390/nano11071733

**Published:** 2021-06-30

**Authors:** Andrea Rossi, Marco Zannotti, Massimiliano Cuccioloni, Marco Minicucci, Laura Petetta, Mauro Angeletti, Rita Giovannetti

**Affiliations:** 1 School of Science and Technology, Chemistry Division, University of Camerino, 62032 Camerino, Italy; andrea.rossi@unicam.it (A.R.); marco.zannotti@unicam.it (M.Z.); laura.petetta@unicam.it (L.P.); 2 School of Biosciences and Veterinary Medicine, University of Camerino, 62032 Camerino, Italy; massimiliano.cuccioloni@unicam.it; 3 School of Science and Technology, Physics Division, University of Camerino, 62032 Camerino, Italy; marco.minicucci@unicam.it

**Keywords:** colorimetric sensor, silver nanoparticles, self-assembly functionalization, 11-Mercaptoundecanoic acid, Nickel detection

## Abstract

Silver nanoparticles (AgNPs) can be used as a surface plasmon resonance (SPR) colorimetric sensor; the correlation between the SPR phenomenon and the aggregation state of nanoparticle allows the real-time detection of a target molecule. Surface functionalization of NPs with proper molecular baits is often performed to establish the selectivity of the sensor. This work reports on the synthesis of AgNPs under reducing conditions and on the functionalization thereof with mercaptoundecanoic acid (11-MUA). UV-VIS Spectroscopy confirmed the formation of AgNPs, eliciting a surface plasmon absorption band (SPAB) at 393 nm that shifted to 417 nm upon surface coating. Dynamic light scattering was used to investigate the surface coatings; moreover, pelleted AgNPs@11MUA nanoparticles were characterized by scanning electron microscopy (SEM), energy dispersive X-ray analyzers (EDX), and infrared spectroscopy to corroborate the presence of 11MUA on the surface. Most interestingly, the resulting AgNPs@11MUA selectively detected micromolar levels of Ni^2+^, also in the presence of other cations such as Mn^2+^, Co^2+^, Cd^2+^, Cu^2+^, Zn^2+^, Fe^2+^, Hg^2+^, Pb^2+^, and Cr^3+^.

## 1. Introduction

The occurrence of heavy metals and their compounds in the environment is the result of both anthropic and natural factors. In fact, heavy metals largely accumulate in the soil and in ground water due to a variety of human activities (e.g., mining, smelting, electroplating, and other industrial processes) and natural weathering of their parent materials [1].

In particular, metals such as Cd, Cr, As, Hg, Pb, Cu, Zn, and Ni are considered toxic, given their persistence in the environment. Consequently, several major international government institutions, in particular the United States Environmental Protection Agency (USEPA), listed them among the priority control pollutants [2,3].

Among these, nickel has attracted special attention not only because of its toxicity, but also because of its widespread use as a catalyst in many industrial processes and its high occurrence in water effluents, where it occurs mostly as divalent cation [4]. Most importantly, nickel was detected (even if at low concentration) in several foods derived either from animal or vegetable sources, with the consequent increase in the risk of toxicity for humans. In fact, exposure to nickel has been demonstrated to be a potential triggering event in pneumonitis, dermatitis, asthma, disorders of central nervous system, and cancer of the nasal cavity or lungs [5].

In general, the quantification of heavy metals involves the application of established techniques, such as wet chemical analyses (e.g., gravimetric, titrimetric, colorimetric, etc.), inductively coupled plasma/atomic emission spectrometry (ICP/AES), inductively coupled plasma/mass spectrometry (ICP/MS), or atomic absorption spectroscopy (AAS) [6].

Despite of offering the adequate sensitivity and specificity, these techniques are often time-consuming, cumbersome, expensive, and require properly trained personnel. In this perspective, several methods have been developed to cope with the increasing demand for the rapid (and possibly user-friendly) detection of metals.

In particular, colorimetric sensors attracted special attention, given their common use in the detection of analytes such as proteins, organic compounds, peptides, nucleic acids, toxic gases, moisture, and water pollutants [7]. Thanks to their ease of use and affordability, colorimetric sensors represent a valuable analytical screening tool since they provide rapid (qualitative) results even in the absence of expensive instrumentation and highly qualified operators. Conversely, these “instrument-free” colorimetric sensors cannot fully meet the criteria of adequate accuracy and sensitivity, which is different from spectroscopic and chromatographic techniques (because the high background noise and the subjectivity of color perception from naked-eye observation). To address these issues, colorimetric sensors are generally coupled with digital imaging techniques [8,9].

In this context, silver and gold nanoparticles are gaining increasing attention [10] for their application in the development of sensors. In fact, because of their peculiar physical-chemical properties, nanoparticles are widely used in colorimetric sensors, exploiting the surface plasmon absorption phenomenon that arises from the collective oscillation of electrons at the interface between the metal and a dielectric [11].

The color of nanoparticles depends on their shape, dimension, composition, and dielectric constant [12], and it changes in the state of aggregation of nanoparticles; the interparticle distance between aggregates causes a shift in the surface plasmon absorption band (SPAB) with respect to monodisperse ones [13,14]. This dependence of SPAB on the state of aggregation is the principle for the detection of chemical substances by colorimetric sensors based on nanoparticles [15].

The functionalization of the nanoparticle surface is a common procedure used to increase/establish the sensor selectivity for a candidate analyte in a matrix containing other chemicals sharing similar properties [16]. Several classes of (bio)molecules can be used as NP functionalizing agents, such as proteins [17], nucleic acids [18], antibody [19], ammino acids [20,21], polymers [22,23], salts [24], and surfactant [25,26], some of these being able to form an electrostatic interaction with NPs [27,28,29,30], and some others forming a covalent bond with the NPs’ surface [31,32].

In particular, alkanethiols are often employed because of their ability to interact with the surface of the metal nanoparticles via their -SH tail [33,34,35]. Among alkanethiols, mercaptoundecanoic acid (11MUA) is an established stabilizer of NPs (silver NPs in particular) in alkaline solution [36,37,38,39,40].

In this work, we report on the synthesis and characterization of silver nanoparticles, on their functionalization with 11MUA, and (for the first time) on the development of a colorimetric sensor based on 11MUA-modified AgNPs for the detection of Ni^2+^ ions in the presence of other metal ions, such as Mn^2+^, Co^2+^, Cd^2+^, Co^2+^, Zn^2+^, Hg^2+^, Fe^2+^, Pb^2+^, and Cr^3+^.

## 2. Materials and Methods

### 2.1. Materials

Silver nitrate (AgNO_3_), sodium borohydride (NaBH_4_), mercaptoundecanoic acid (11MUA), NiCl_2_, CoCl_2_, ZnCl_2_, CuCl_2_, MnCl_2_, CdCl_2_, FeCl_2_, HgCl_2_, PbCl_2_, and CrCl_3_ were purchased from Sigma-Aldrich (St. Louis, MO, USA), and used without further purification. All the glassware was washed with boiling aqua regia before being used. All the solutions were prepared using ultrapure water (18.2 µS/cm).

Plots, fittings, and statistical analysis were performed by using MatLab R2020b (The MathWorks, Inc, Natick, MA, USA).

### 2.2. Synthesis of AgNPs and AgNPs@11MUA

Silver nanoparticles (AgNPs) were obtained by chemical reduction with NaBH_4_, according to a modification of the method of Mulfinger et al. [27]. The synthetic procedure was optimized by testing different conditions of temperature, stirring time/rate, and Ag-to-NaBH_4_ molar ratio. Optimal colloidal self-stability was achieved by adding 1 mL of 0.01 M NaBH_4_ to 50 mL of 2.15 mM AgNO_3_ ([AgNO_3_]/[NaBH_4_] = 1:5) in ultrapure water and left stirring for 30 min at 0 °C. The shift from colorless-to-yellow in the color of the solution indicated the formation of NPs [27]. AgNPs’ surface was functionalized by adding 104 µL of 11MUA (0.0103 M in pH 9 NaOH solution) to the colloidal solution and stirred for 24 h. Upon functionalization, the AgNPs@11MUA suspension was dialyzed using a Spectra/Por 3 dialysis membrane, 3500 Da cut-off, (Campton, CA, USA) to remove the uncoupled 11MUA. The dialyzed solution was then lyophilized to obtain the nanoparticle pellet to be used for the characterization.

### 2.3. Characterization of AgNPs and AgNPs@11MUA

UV-VIS measurements were performed by using Cary 8454 Diode Array System spectrophotometer (Agilent Technologies, Santa Clara, CA, USA).

Field emission scanning electron microscopy (FE-SEM, Sigma 300, Zeiss, Gina, Germany) operating at 7 kV, equipped with energy dispersive X-ray spectroscopy (EDX, Quantax, EDS, Bruker, Billerica, MA, USA), was used to evaluate the morphology of AgNPs and AgNPs@11MUA. Samples were prepared in the same manner reported in a previous study [11].

Infrared spectroscopy characterization was performed by using Perkin-Elmer System 2000 FT-IR instrument (Waltham, MA, USA). IR spectra of AgNPs and AgNPs@11MUA pellets were collected from 4000 to 10 cm^−1^.

Dynamic light scattering was used to investigate the effect of 11MUA coating and the formation of lattice upon Ni^2+^ addition; measurements were performed on a Malvern Zetasizer nano S device (Malvern Instruments, Worcestershire, UK) equipped with a back-scattered light detector operating at 173°.

### 2.4. Colorimetric Sensing Applications

Stock solutions of different ions (Ni^2+^, Cu^2+^, Zn^2+^, Cd^2+^, Co^2+^, Mn^2+^, Fe^2+^, Pb^2+^, Hg^2+^, and Cr^3+^) with a concentration of 0.1 mM were prepared from the corresponding chlorinated salts. For a general method, 1 mL of AgNPs@11MUA with 1 mL of ultrapure water was placed in a cuvette and titrated with stepwise additions of 10 µL of individual metal ion solutions. SPAB spectra were recorded and compared at each step of titration after 1 min of stirring.

## 3. Results

### 3.1. Characterization of AgNPs and AgNPs@11MUA

#### UV-VIS Characterization

**Figure 1:**
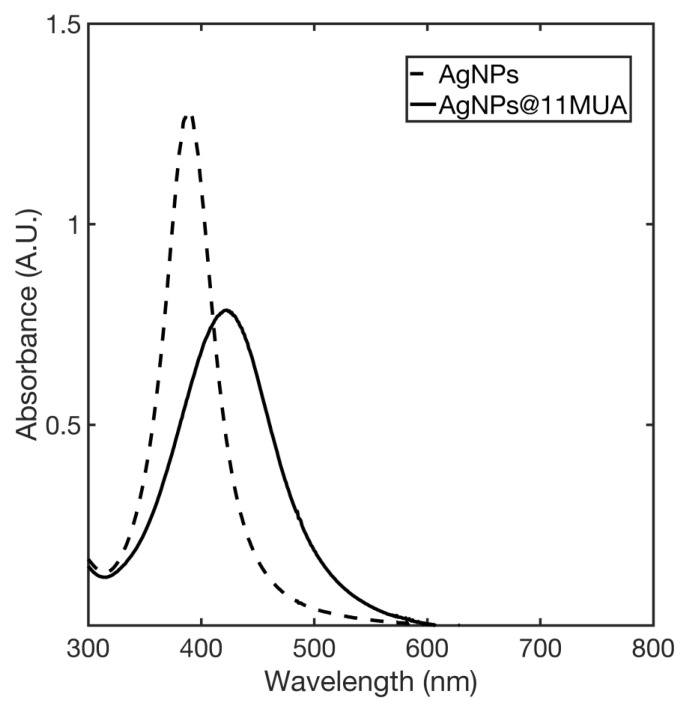
SPAB of AgNPs (dashed line) and AgNPs@11MUA (solid line).

The formation of AgNPs was confirmed by the presence of pale-yellow color solution, which became more intense upon functionalization with 11MUA. UV-VIS spectra of synthetized AgNPs and AgNPs@11MUA are reported in Figure 1. Spectra show strong absorption at 393 nm for AgNPs, whereas the SPAB is red shifted to 417 nm in the AgNPs@11MUA sample as the result of the interaction between AgNPs’ surface with 11MUA [41,42,43,44]. Borohydride ions (BH_4_^−^) [45] on the surface of AgNPs and 11MUA molecules on the surface of AgNPs@11MUA [35] electrostatically stabilized AgNPs and AgNPs@11MUA, respectively.

### 3.2. Dynamic Light Scattering Characterization of AgNPs and AgNPs@11MUA

Dynamic light scattering analyses were performed on AgNPs, AgNPs@11MUA, and AgNPs@11MUA after addition of Ni^2+^ (see Supplementary Materials Figures S4–S6). DLS results, summarized in Table 1, show an average diameter size (Z-Ave) of 27.55 ± 3.77 nm for AgNPs sample and the average diameter size increasing to 173.13 ± 1.66 nm after functionalization with 11MUA. After addition of Ni^2+^, the average diameter size rapidly increases up to 6123.33 ± 349.01 nm due to the formation of AgNPs@11MUA lattice.

### 3.3. Scanning Electron Microscopy Characterization of AgNPs and AgNPs@11MUA

**Figure 2:**
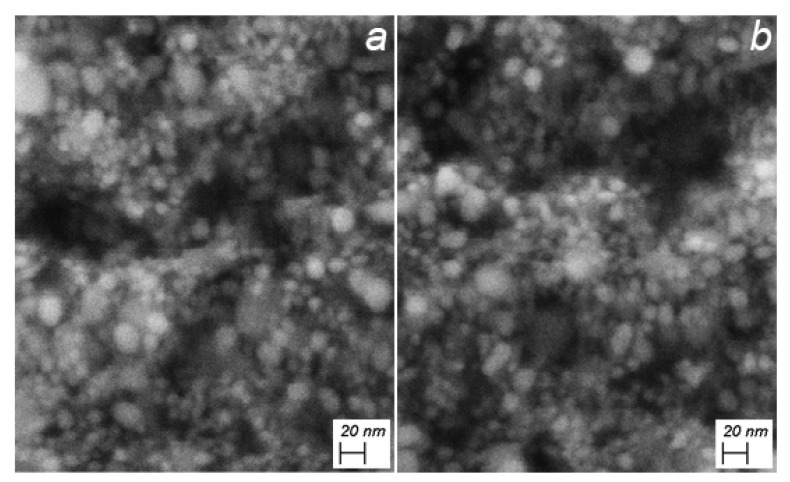
Morphology of (**a**) AgNPs and (**b**) AgNPs@11MUA. Operative condition: 7.00 kV, WD = 3.7 mm and Mag = 400.00 kX.

Morphological analysis by SEM demonstrated that the obtained sample showed a spherical distribution of AgNPs having a maximum diameter size around 20 nm (Figure 2a). Upon 11MUA functionalization, the morphology and dimension remains almost the same, displaying a spherical distribution [38] (Figure 2b).

**Figure 3:**
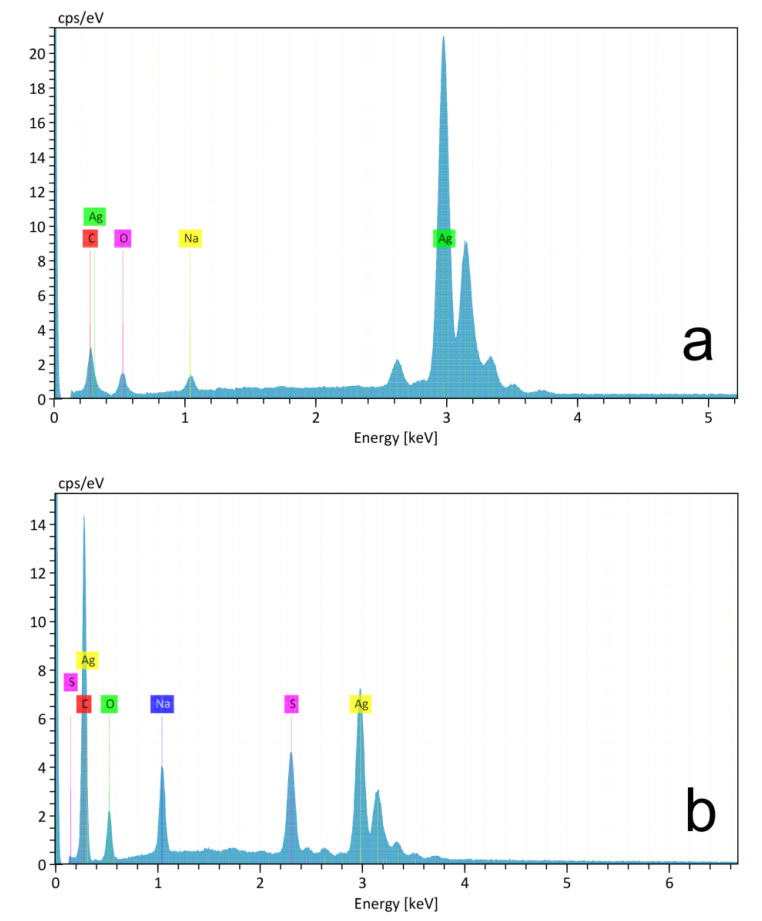
**(a)** EDX analysis of AgNPs and **(b)** AgNPs@11MUA sample.

EDX spectra of AgNPs and AgNPs@11MUA are reported in Figure 3a,b, respectively. After dialysis, the EDX spectrum of AgNPs@11MUA revealed the presence of sulfur in the AgNPs@11MUA sample, indicating a good functionalization process.

### 3.4. Infrared Spectroscopy Characterization of AgNPs and AgNPs@11MUA

**Figure 4:**
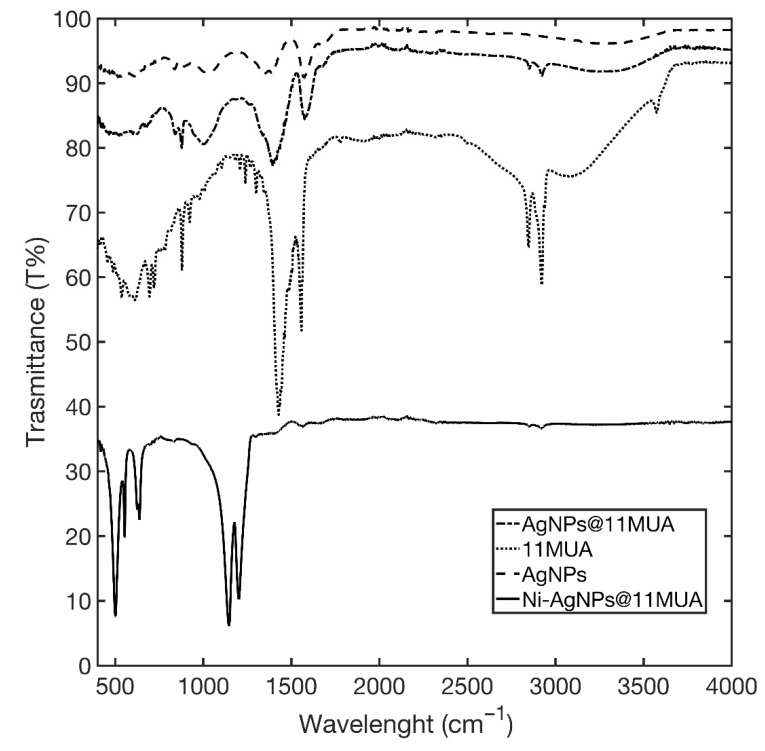
IR spectra of the AgNPs@11MUA (solid line), 11MUA (line dash point), and AgNPs (dashed line).

The infrared spectrum (IR) of 11MUA is characterized by two strong bands at 2917.77 cm^−1^ and 2850.27 cm^−1^ of C-H stretching that were also present in the spectrum of AgNPs@11MUA (Figure 4). Additionally, a weak and broad band in the region 2600–2550 cm^−1^ of S-H stretching of thiol group was observed only in the 11MUA spectrum. The band corresponding to the thiol group was not present in AgNPs@11MUA, indicating a good functionalization process. Moreover, the band of C=O stretching was observed at 1710.55 cm^−1^, the broad band in the region 2900–3300 cm^−1^ corresponds to the O-H stretching of carboxylic acid, the band at 1458.37 cm^−1^ is related to the O-H bending, while the band at 1301.72 cm^−1^ is in accord with C-O stretching [46]. Ni^2+^ coordination caused a strong shift of the bands corresponding to carboxylic acid from 1458.37 and 1301.72 cm^−1^ to 1203.10 and 1147.60 cm^−1^, respectively, and the disappearance of the band at 1710.55 cm^−1^. Additionally, the new bands in the region at around 580 cm^−1^ being attributed to the Ni-O stretching vibration mode [47].

### 3.5. Effect of Metal Ions on AgNPs

**Figure 5:**
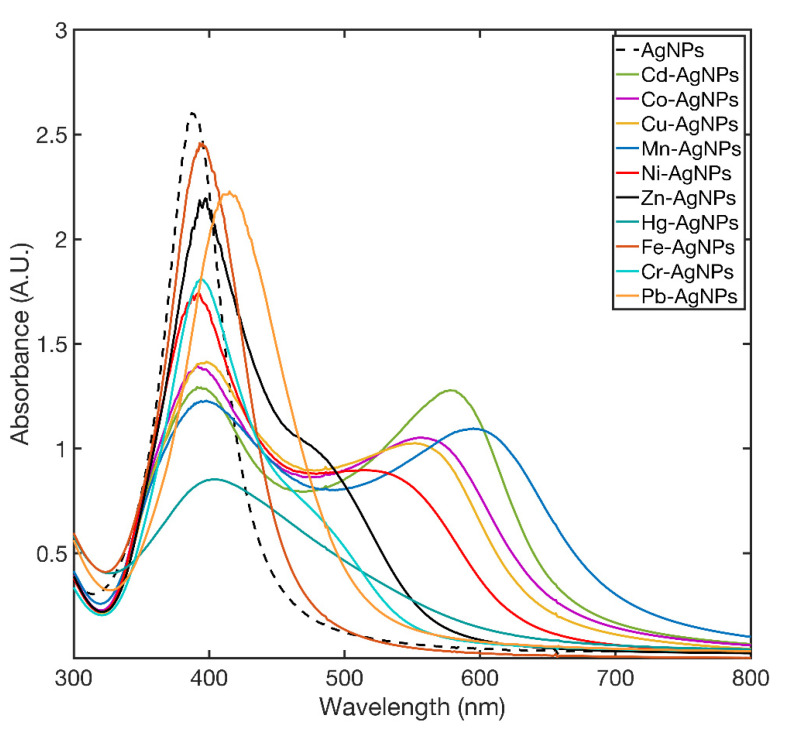
SPAB spectra of AgNPs recorded at 5 min after the addition of metal ions.

To test the AgNPs behavior in the absence of 11MUA, 10 μL of Co^2+^, Mn^2+^, Cd^2+^, Cu^2+^, Zn^2+^, Fe^2+^, Hg^2+^, Pb^2+^, Cr^3+^, and Ni^2+^ stock solutions (0.1 mM) was added to the AgNPs dispersion. Figure 5 shows the UV-VIS spectra of the AgNPs collected 5 min after the addition of metal ions. As it can be observed, the typical spectra of aggregated AgNPs were obtained after the addition of each metal ion; all the AgNPs fully aggregated after 15 min.

### 3.6. Effect of Metal Ions on AgNPs@11MUA

UV-VIS spectroscopy was used to evaluate the optical properties of AgNPs@11MUA and of its complexes thereof with Co^2+^, Mn^2+^, Cd^2+^, Cu^2+^, Zn^2+^, Fe^2+^, Hg^2+^, Pb^2+^, Cr^3+^, and Ni^2+^ ions.

**Figure 6:**
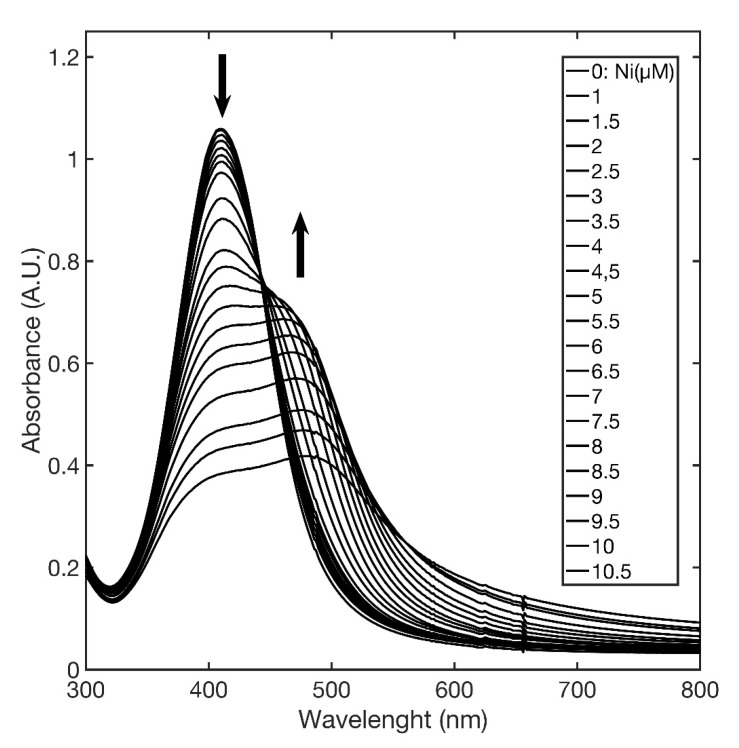
Changes in SPABs upon addition of increasing concentration of Ni^2+^ to AgNPs@11MUA.

Specifically, AgNPs@11MUA showed a single SPAB at 417 nm, and the addition of Ni^2+^ caused a color change from yellow to purple, with the appearance of a second SPAB at 477 nm. In fact, Figure 6 shows that upon titration of AgNPs@11MUA with Ni^2+^ (in the range 0–10 µM), the SPABs at 417 and 477 nm decreased and increased in intensity with Ni^2+^, respectively.

**Figure 7:**
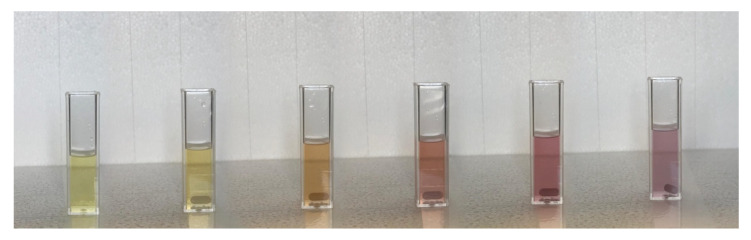
Color changes of AgNPs@11MUA upon addition of increasing concentration of Ni^2+^.

Figure 7 shows the change in the color of the AgNPs@11MUA suspension upon titration with Ni^2+^.

**Figure 8:**
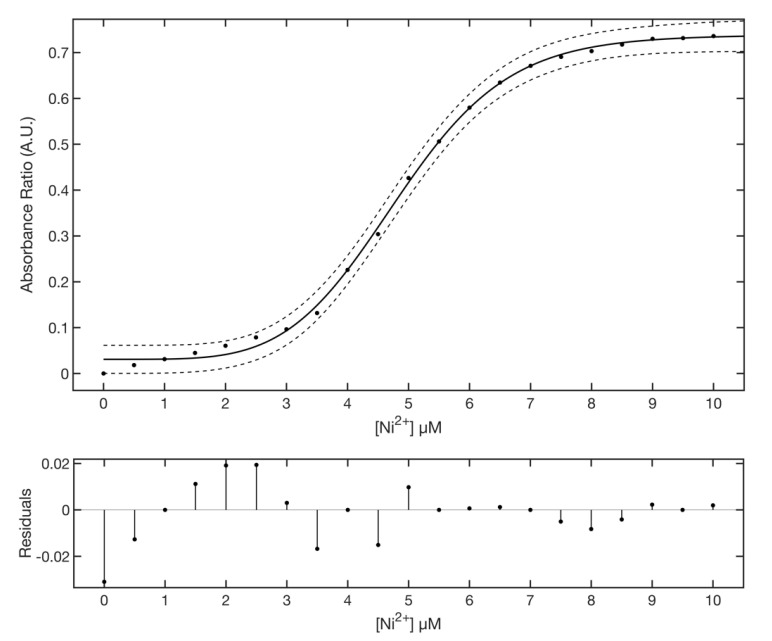
Upper panel: dependence of absorbance ratio from Ni^2+^ concentration. Fit of raw data for Ni-AgNPs@11MUA to Equation (1) (solid line). The 95% confidence interval is shown (dashed line). Lower panel: residuals plot of the fitting.

Titration curves clearly show the occurrence of an isosbestic point at about 445 nm that splits the SPAB in two zones and supports a mechanism of direct interconversion between AgNPs@11MUA and (AgNPs@11MUA)_n_-(Ni^2+^)_m_ cluster [48]; the individual contribution in absorbance of the two SPABs was evaluated by deconvoluting the spectra with a lognormal equation [49,50,51] (Figure S1, Supplementary Materials). Titration curves were obtained by plotting the absorbance ratio at 417 and 477, respectively, as a function of Ni^2+^ concentration (Figure 8).

**Figure 9:**
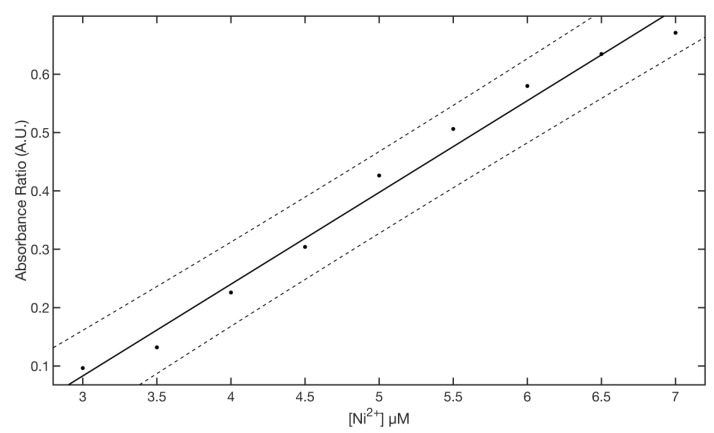
Linear region of the sensor ([Ni^2+^] = 3–7 µM).

Titration data were fitted to a five parameter logistic function [52],

(1) $$f\left(x\right)=d+\frac{\left(a-d\right)}{\left({\left(1+{\left(\frac{x}{c}\right)}^{b}\right)}^{g}\right)}$$

where *a* and *d* parameters control the position of the lower and upper asymptotes, *b* and *g* are related to the rapidity of the curve transition between the two asymptotes, and *c* indicates the position of the transition. Fitting results gave an *R* square of 0.9981 with an SSE of 0.0027. The best fitted values for the parameter to 95% confidence bounds were: *a* = 0.5365 ± 0.0125, *b* = 4.544 ± 0.984, *c* = 6.742 ± 0.422, *d* = 0.7387 ± 0.0255, *g* = 2.011 ± 0.503. Figure 8 lower panel shows the residuals plot of the fitting. The linear region of the titration curve (3–7 µM) was fitted with a linear equation $f\left(x\right)=m*x+q$, where *m* is the slope and *q* the intercept (Figure 9).

**Figure 10:**
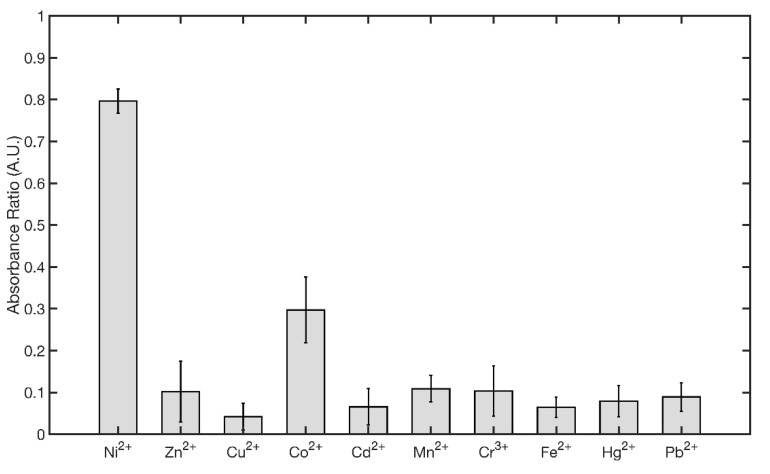
Comparison of the absorbance ratios of AgNPs@11MUA after addition of 7.5 µM of different metal ions.

The best fitted values for the parameter to 95% confidence bounds were *m* = 0.1572 ± 0.0171 and *q* = −0.3885 ± 0.0095. Fitting results gives an R square of 0.9853 with an SSE of 0.0055. The limits of detection (LOD) and limits of quantitation (LOQ) were calculated from the calibration curve as 3 *σ/m* and 10 *σ/m* [53], where *σ* is the standard deviation of intercept, and *m* is the slope of the regression curve fitting the data points in the linear range (3–7 µM). The obtained value of LOD and LOQ were 2.15 µM and 7.16 µM, respectively. Although presenting a similar general behavior, all other metal ions of interest were associated with minor-to-negligible changes in UV-VIS spectra (Figure S2, Supplemental Materials). For comparative purposes, the responses at 7.5 µM of each metal ion are shown in the bar plot reported Figure 10 and in UV-VIS spectra in Figure S3.

To establish the selectivity of the AgNPs@11MUA sensor, the cross-reactivity values for each metal ion were calculated as:

(2) $$CR\%=\frac{AbsRatio_{Metal\;ion@7.5µM}}{AbsRatio_{Ni^{2+}@7.5µM}}\times 100$$

The values are summarized in Table 2.

A possible rationale behind the observed behavior is that only the association of NPs into larger clusters with bridging Ni^2+^ ions (that results in the formation of a “superlattice” [54]) can cause the shift in SPAB. On such a premise, the first part of the curve in Figure 8 represents the slow formation of a small lattice of AgNPs@11MUA in the presence of low concentration of Ni^2+^ (the lag phase, low-sensitivity region). Next, as the Ni^2+^ concentration increases, it triggers the aggregation of a higher number of additional nanoparticles. This phenomenon results in a bigger lattice (the “superlattice”) that is associated with greater change in the absorbance spectrum and higher sensitivity of the sensor.

Next, upon further increase in Ni^2+^ concentration, the NPs-Ni^2+^ cluster reaches a critical mass and the superlattice collapses.

**Figure 11:**
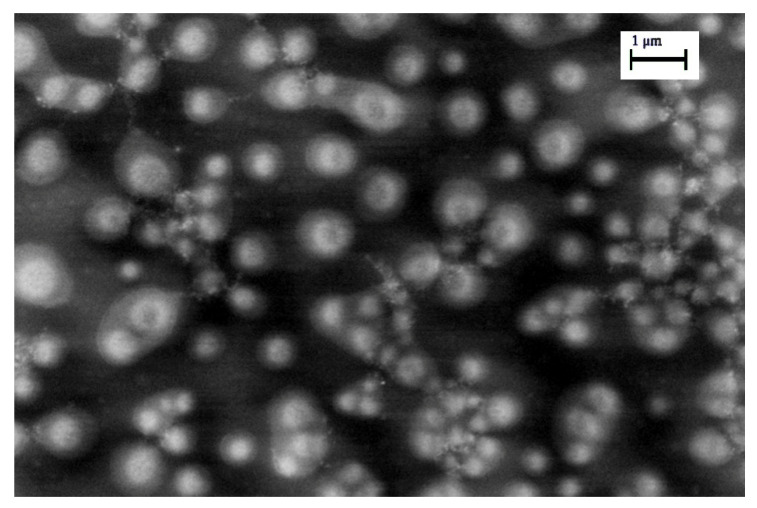
Morphology of AgNPs@11MUA after addition of 7.5 µM of Ni^2+^. Operative condition: 7.00 kV, WD = 1.4 mm and Mag = 19.17 kX.

In order to obtain additional information, SEM measurements were performed (as reported in Figure 11) that clearly show early stage clusters of AgNPs collapsing at Ni^2+^ concentration of 7.5 µM; it is evident the formation of lattice consists of NP subclusters, each being about a micron size.

**Figure 12:**
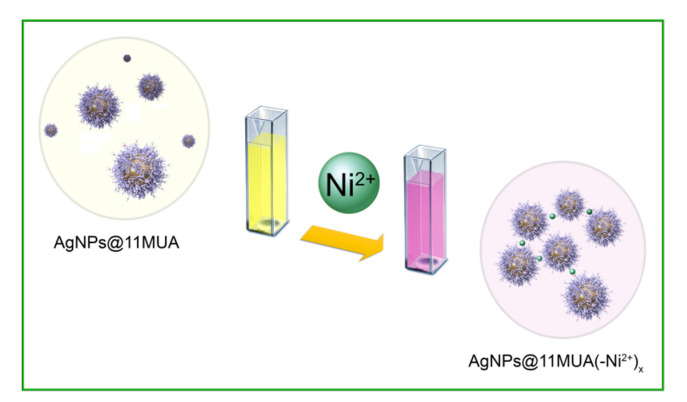
Schematic clustering process in the in the interaction of Ni^2+^ ions with AgNPs@11MUA.

A schematic representation of the clustering process between AgNPs@11MUA and Ni ions is reported in Figure 12.

Given the sigmoidal shape of the titration curve, the interaction of AgNPs@11MUA with Ni^2+^ can be considered a random-like process that is regulated by apparent cooperativity, i.e., the binding of the first Ni^2+^ ion to the AgNPs@11MUA is associated with a structural modification that results in a more favorable binding of subsequent ones. Specifically, the increase in Ni^2+^ concentration is associated with higher clustering rate, faster appearance, and increase in intensity of the second SPAB, and consequent faster response of the sensor. Conversely, the increasing steric hindrance and the critical size of NPs-Ni^2+^ clusters represent the limiting factors of the sensor. Therefore, by adapting Hill’s equation [55] to our system, it was possible to obtain quantitative information on the mechanism of the formation of a superlattice of nanoparticles. AgNPs@11MUA can be considered as a macromolecule with n binding sites, *n* corresponding to the number of accessible COO^−^ groups of surface-bound 11MUA. Hill’s equation relates the saturation to unsaturation ratio (number of occupied COO^−^ to the number of free COO^−^ ratio) to the concentration of the ligand (Ni^2+^).

**Figure 13:**
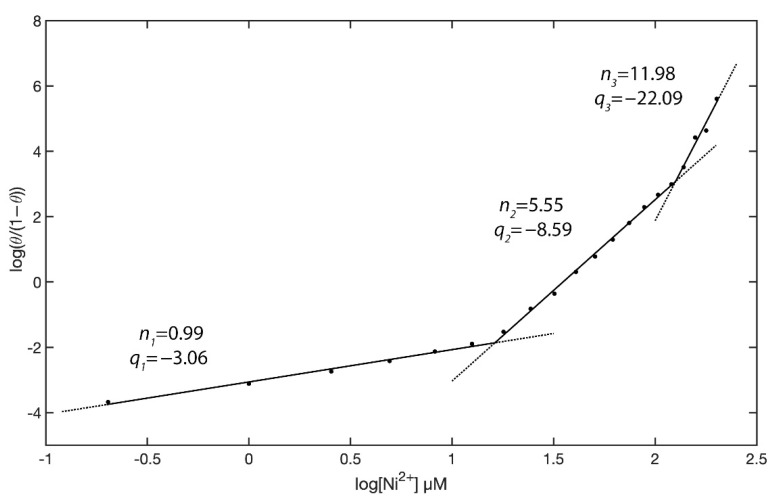
Hill plot for at AgNPs@11MUA binding to increasing concentrations of Ni^2+^. Local linear fits to Equation (3) are displayed as solid lines.

In our system the saturation (θ) can be calculated as Equation (3):

(3) $$\theta =\frac{Abs\;Ratio_{i}}{Abs\;Ratio_{max}}$$

where *Abs Ratio_i_* is the value obtained for the generic concentration of Ni^2+^, and *Abs Ratio_max_* is the value obtained at asymptotically high concentrations of Ni^2+^ (corresponding to “*d*” parameter from Equation (1). Consequently, unsaturation can be calculated as 1 − θ. Hill’s equation can be expressed as in Equation (4):

(4) $$log\left(\frac{\theta }{1-\theta }\right)=nlog\left[Ni^{2+}\right]-logK_{D}$$

where *n* is the Hill’s coefficient and indicates the average number of bridging Ni^2+^ ions between two AgNPs@11MUA (i.e., the number of AgNPs@11MUA molecules forming the cluster), and *K_D_* is the equilibrium constant for AgNPs@11MUA clustering. The Hill plot (Figure 13) displays three different linearity zones, with individual slopes being n_1_ = 0.99, n_2_ = 5.55, and n_3_ = 11.98.

These data suggest the existence of a different average number of bridging Ni^2+^ (-Ni^2+^-) between AgNPs@11MUAs at different concentrations of free Ni^2+^. Specifically, (-Ni^2+^-) = 1 being prevalent for Ni^2+^ concentrations in the range 0–3 µM, (-Ni^2+^-) = 5/6 being prevalent for Ni^2+^ concentrations in the range 3–7 µM, and (-Ni^2+^-) = 12 being prevalent for Ni^2+^ concentrations in the range 7–10 µM.

Most interestingly, the intermediate zone corresponded to the linearity range of the proposed AgNPs@11MUA sensor, with the AgNPs@11MUA cluster linked by (-Ni^2+^-) = 5/6 thus being inferred as the ideal sensing cluster. At higher concentrations of Ni^2+^, the NPs cluster further increased in size but were likely to precipitate, causing the superlattice (and the surface plasmon absorption) to collapse.

## 4. Conclusions

In this paper, we reported on the synthesis and functionalization of silver nanoparticles, which can be exploited as a sensor for metal ions. The nanoparticles were obtained by chemical reduction with NaBH_4_, then they were functionalized with bifunctional 11MUA exploiting the affinity between thiol groups and the silver NPs surface. Upon chemical and structural characterization, these nanoparticles have been demonstrated to be spherical, with a maximum diameter size around 20 nm and a successful coating with 11MUA. Furthermore, these NPs were shown to associate into larger clusters in the presence of divalent cations that act as bridges between COO^−^ groups of 11MUA, eventually forming a “superlattice”. Interestingly, this aggregation phenomenon led to a major change in optical properties of the NPs suspension, consisting of the progressive decrease in the intensity of the main SPAB at 417 nm (associated with the “monomeric” functionalized nanoparticle) and the appearance of a secondary SPAB at 477 nm, which increased in intensity with metal ions concentration. The higher rate in NPs clustering upon the addition of Ni^2+^ with respect to the other metal ions of interest (and the consequent faster change in absorbance at 417 nm and 477 nm) allowed the use of AgNPs@11MUA as a rapid colorimetric sensor for the qualitative detection of micromolar levels of Ni^2+^ ions in water with acceptable selectivity.

## Figures and Tables

**Table 1:** DLS results of AgNPs, AgNPs@11MUA, and Ni-AgNPs@11MUA, respectively.

Sample	Replicate	Z-Ave (d.nm) ^a^	PdI ^b^
AgNPs	1	29.04	0.619
	2	23.26	0.78
	3	30.35	0.495
AgNPs@11MUA	1	171.6	0.286
	2	172.9	0.253
	3	174.9	0.271
Ni-AgNPs@11MUA	1	5760	1.000
	2	6456	1.000
	3	6154	1.000

^a^ average diameter size; ^b^ Polydispersity index (PdI).

**Table 2:** Cross reactivity values for each metal ions calculated by Equation (2).

Metal Ions	CR%
Ni^2+^	100
Zn^2+^	13
Cu^2+^	6
Co^2+^	37
Cd^2+^	8
Mn^2+^	14
Cr^3+^	13
Fe^2+^	8
Hg^2+^	10
Pb^2+^	11

## Data Availability

The completed data of this study are available from the corresponding author, upon reasonable request.

## Supplementary Materials

The following are available online at https://www.mdpi.com/article/10.3390/nano11071733/s1, Figure S1: UV-VIS signal deconvolution of (a) AgNPs@11MUA and (b) AgNPs@11MUA after addition of 7.5 µM of Ni^2+^ using Fityk software, Figure S2: Comparison of titration curves of AgNPs@11MUA with increasing concentration of the metal ions of interest, Figure S3: UV-VIS spectra of AgNPs@11MUA after addition of 7.5 µM of Co^2+^, Mn^2+^, Cd^2+^, Cu^2+^, Zn^2+^, Fe^2+^, Hg^2+^, Pb^2+^, Cr^3+^, and Ni^2+^ ions, Figure S4: DLS measurements on AgNPs, Figure S5: DLS measurements on AgNPs@11MUA, Figure S6: DLS measurements on AgNPs@11MUA after addition of Ni^2+^.
