# Supplementary material for: Silver Nanoparticle-Based Sensor for the Selective Detection of Nickel Ions
Source: Nanomaterials (Basel). 2021 Jun 30;11(7):1733. doi: 10.3390/nano11071733 (PMC8308118; doi:10.3390/nano11071733)
Supplement: Supplementary file 1 [file nanomaterials-11-01733-s001.zip › nanomaterials-1257301-supplementary.pdf]

SUPPLEMENTARY MATERIALS

Silver Nanoparticle-Based Sensor for the Selective Detection of Nickel Ions

Andrea Rossi [1], Marco Zannotti [1], Massimiliano Cuccioloni[2], Marco Minicucci [3], Laura Petetta [1], Mauro Angeletti [2,*] and Rita Giovannetti [2,*]

[1] School of Science and Technology, Chemistry Division, University of Camerino, 62032 Camerino, Italy; andrea.rossi@unicam.it (A.R.); marco.zannotti@unicam.it (M.Z.); laura.petetta@unicam.it (L.P.)

[2] School of Biosciences and Veterinary Medicine, 62032 Camerino (MC), Italy; massimiliano.cuccioloni@unicam.it (M.C.)

[3] School of Science and Technology, Physics Division, University of Camerino, 62032 Camerino, Italy; marco.minnicucci@unicam.it (M.M.)

* rita.giovannetti@unicam.it (R.G.); mauro.angeletti@unicam.it (M.A.)

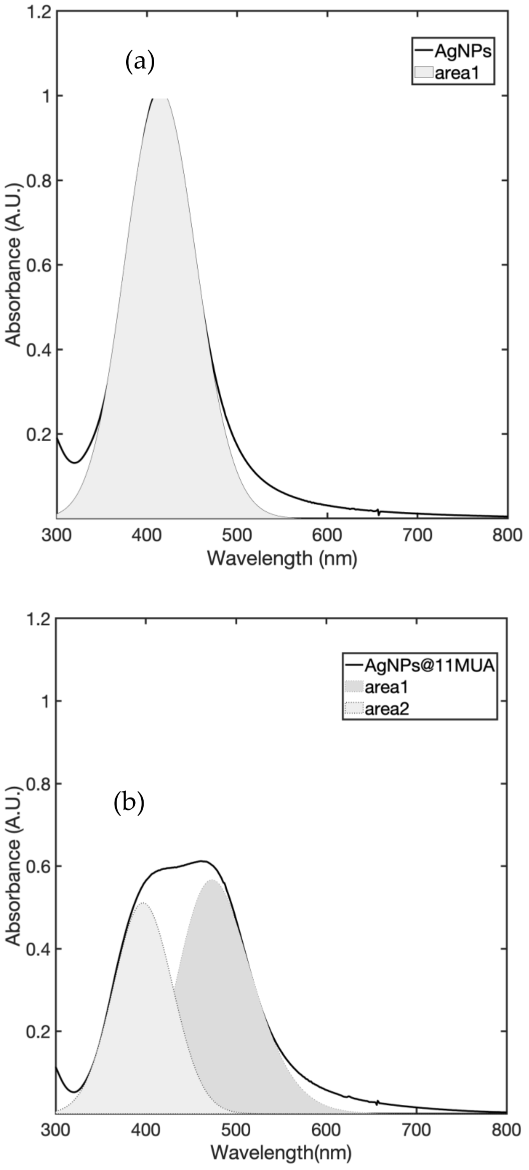

Figure S1. UV-VIS signal deconvolution of (**a**) AgNPs@11MUA and (**b**) AgNPs@11MUA after addition of 7.5 μM of Ni^{2+} using FityK software.

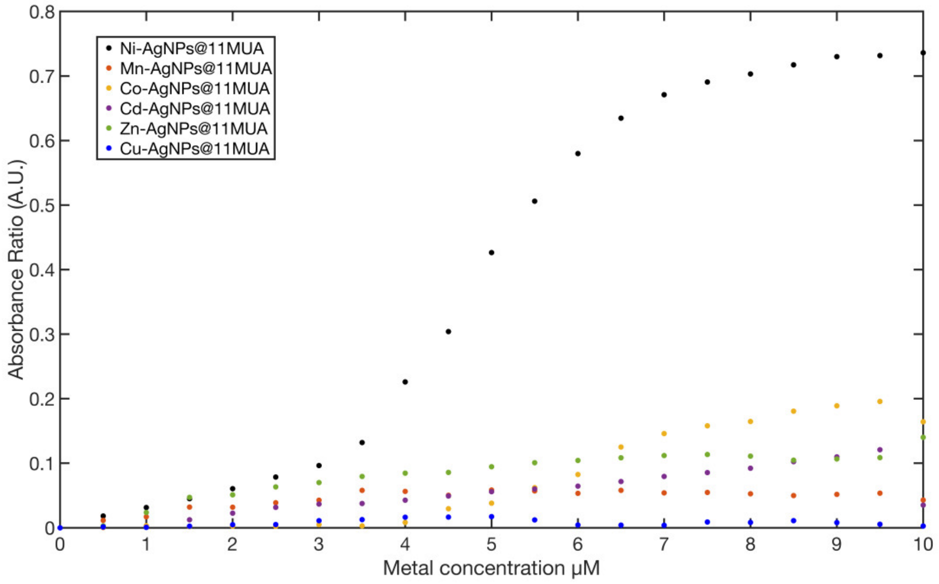

Figure S2. Comparison of titration curves of AgNPs@11MUA with increasing concentration of the metal ions of interest.

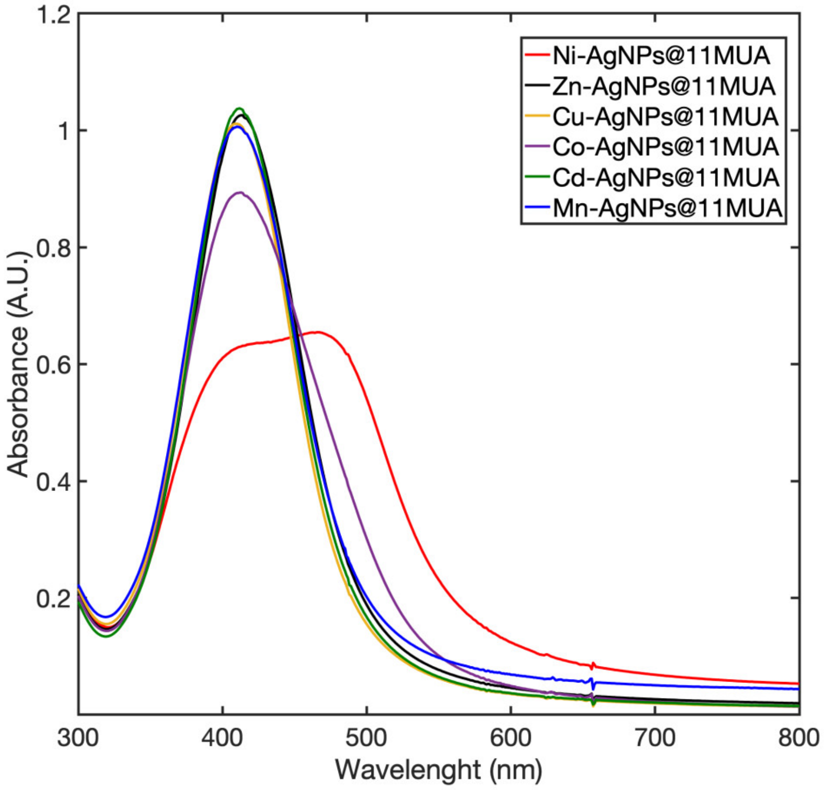

Figure S3. UV-VIS spectra of AgNPs@11MUA after addition of 7.5 μM of Co^{2+}, Mn^{2+}, Cd^{2+}, Cu^{2+}, Zn^{2+}, and Ni^{2+} ions.
